# A Medico-Legal Treatise on Homicide by External Violence

**Published:** 1838-07-01

**Authors:** 


### FORENSIC MEDICINE.

### A Medico-legal Treatise on Homicide by External Violence—with an Account of the Circumstances which Modify the Medico-legal Characters of Injuries and Exculpatory Pleas. By *Alexander Watson, Esq.*, F.R.S.E., and one of the Surgeons of the Royal Infirmary, &c., &c., &c. Edinburgh and London 1837, pp. 355.

In our number for January we reviewed a volume from the Irish press, on the difficult subject of pregnancy, its signs and symptoms. We are now about to notice a work from the Scottish press on a matter of equal importance—homicide.

We had then the pleasure of recommending Dr. Montgomery's volume to general notice, and have no less pleasure now in introducing Mr. Watson to our readers. And thus in two successive articles on medical jurisprudence to make the extremes meet of the beginning and the termination of life.

By Mr. Watson the subject is treated of under twelve heads, and, in order to facilitate the analysis of his work, we shall notice each separately.

#### I. General Remarks on Medical Jurisprudence.

Legal, or forensic medicine, is altogether a science of facts; it has nothing, and ought to have nothing, to do with opinions. In its application to practical purposes, its true object must ever be, the development of truth, and that alone.

The value of medical testimony will therefore be always in proportion to the medical witness's acquaintance with the facts of his profession—and, the most accomplished practitioner, will ever bear the most respectable evidence, in all the cases which ordinarily occur to summon him from the active duties of his profession, to wile away his time in courts of law, listening while the Belials of our earth exert their eloquence on whatever side hired, to "make the worse, appear the better cause," until summoned in his turn to add the weight of his assertions, under the solemn sanction of an oath, to the scale of even-handed justice. His duty is then plain—to declare the truth—the whole truth—and nothing but the truth—so help him, God! His competency to declare the truth and not a lie, will mainly, if not wholly, rise out of the perfection of his general professional attainments, whether he may have studied medical jurisprudence distinctly, as a separate branch of the manifold science of medicine, or only made himself master of the several questions to which it gives rise in the ordinary course of his general medical studies.

"My object in examining, or cross-examining witnesses," said a venerable friend to the writer of this review, "is to develop truth. My aim and endeavour in undertaking the cause of a client, is to carry it to a successful issue if it deserve one—and, if my client be clearly unworthy, to maintain for him the right of every man, to be judged only according to law, and not condemned contrary to law." Such is the duty of the advocate. A difficult and a dangerous one—at once artful, complicated, and cunning, must be the efforts of him who undertakes it. How beautifully simple, easy, and unencumbered, on the contrary, is the task assigned to the medical witness. He has only to state facts as he finds them, and leave truth to make its own way in the minds of those who sit as judges. And, the more rigidly he confines himself to facts, and avoids the hazard of opinionating himself, and the indelicacy of meddling with the opinions of others, the better. True, judges sometimes, and advocates always, will endeavour to get at the opinions of medical witnesses. The latter from a very natural desire to strengthen his own case and weaken his adversary's. Facts are facts, and too stubborn to bend or break under a lawyer's tongue, or be pulverized between a lawyer's teeth. Once sworn to, there let them rest, silent yet most eloquent, because all-persuasive;—whereas, opinions may or may not be true. And while, if the facts be admitted, the conclusion from them is certain, argument against them militates nothing—declamation itself is hushed in their presence;—opinions are not so—the witness has one opinion—the lawyer another—the witness may state his opinion but he may not defend it, or declaim upon it—the argument is the lawyer's, and he argues alone, there is none to answer him but a lawyer, who cannot so well as the author of the objections, argue in favor of an opinion of which *he* is not the author—and which to him is altogether extra-professional — perhaps altogether unheard of.

The difficulty is not, how to state, but how to ascertain the facts of any given case. And, unless the witness be morally certain of the truth of his facts;—certain, beyond all possibility of doubt, that their accuracy cannot be impeached, he should hold his peace and not bear witness at all. Not only property, but liberty, and even life itself, to say nothing of that without which prolonged life is only protracted misery—character, each, and all may hang upon his breath—and be saved or forfeited, according as he shall testify.

The use therefore of medical jurisprudence is, not to supersede the knowledge which every man must acquire for himself—from experience, observation, and study. But to distinguish that knowledge which is required for the purposes of justice, and to separate the facts upon which it is based, from the crowd of other facts, which, however valuable, may embarrass but cannot assist investigations partaking as much of a legal as of a medical character.

The qualification of a medical witness to assist coroner, judge, or jury, is knowledge—professional knowledge. The greater his knowledge the better is he qualified. Nevertheless, since medical jurisprudence has only very recently—within the last ten years—been made a *sine quâ non* in the curriculum of a medical education, whether in London, Edinburgh,* or Dublin—and as many persons hold diplomas who never studied the subject at all, we do not agree with Mr. Watson that "medical jurists should be men holding diplomas from some of the legally constituted medical faculties." We, however, fully coincide in what follows :—

"The extent of medical knowledge possessed by a medical jurist cannot be too great. He requires to know accurately the symptoms, morbid phenomena, and the proper treatment of all diseases and injuries, the effects of remedies, of poisons, and of other external agents, upon the body. This is necessary, in order that he may be able to decide correctly on any given case, whether natural disease, poison, injury by violence, the effects of substances used as remedies, or other accidental circumstances, caused death; and in order also that he may be able to judge whether the treatment of the injured person, which had been adopted, was proper, and was applied in conformity to the established principles of the profession, as well as to see that no important measure was omitted which might have saved the life of the deceased." 6.

The evils resulting from the contradictory opinions of medical witnesses, summoned on opposite sides of the same cause, call forth a very indignant but proper remonstrance from Mr. Watson, on the injury such contradictions are apt to do the parties whose interests are at stake—and on the degradation to which they expose the profession itself. But he fails to trace those evils, to their original cause, which is the unconstitutional, and, with all deference be it spoken, the illegal practice of calling upon witnesses to state their opinions—instead of confining them to a simple statement of facts—the truth—the whole truth—and nothing but the truth. Neither judge nor jury have any right to demand of a witness, in what light that witness regards the facts to which he has testified—it is for them to opine—decide—and pronounce judgment accordingly : the witness's duty is done when he has given his evidence—and opinions enter not into the nature of evidence :—all beyond mere truth-telling—and opinions, however valuable in some instances, are not necessarily, nor always, truths—is supererogatory—and may prove fatal to the ends of justice, by biassing the judge, and exercising an undue influence over the jury. For all men are naturally disinclined to labour and averse from responsibility—and if judges can remove from themselves the labour for which they alone are paid, of sifting evidence, and separating the bad from the good, the false from the true, the important from the unimportant—and if jurymen can absolve themselves from the heavy responsibility of "well and truly trying" the case before them, and skulk from the upbraidings of a disquieted conscience to hide them under the

* The Forensic Medicine chair is a little, and only a little older, than the time above stated.

shelter of opinions which ought never to have been given or received in a court of justice—they will very composedly allow the medical witness to do their work for them, to underlye their responsibility—and—to make himself and the profession of which he is a member a "bye-word and a mockery" among men.

Having made these observations upon the subject generally, we proceed to the author.

### II. and III.—General Remarks on Homicide and on Death by Violence.

Homicide admits of four divisions—intentional, or wilful—culpable—unavoidable—and justifiable.

Death by violence, follows upon an injury done to one or other of the sets of organs known as vital, or it may be occasioned by the sympathy subsisting between one set of organs and another; the nervous system being affected by derangements of the circulating system—and both affecting the functions of respiration and digestion to the loss of life from the extent to which they become affected, when, but for such secondary affections, the original injury might have been wholly inadequate to produce a fatal result.

Some injuries are necessarily fatal, from the importance of the organs concerned—from being beyond the reach of art—or, from the magnitude of the injury.

On the other hand, a diseased habit of body, or a deranged condition of parts, may render an injury fatal to one individual, which would be of no moment to another. And, to the same individual, the injuries which at one time would be comparatively harmless, might at another be of the most serious consequence.

The injury, moreover, may be so modified as to occasion the loss of life from inattention, negligence, or unskilfulness, on the part of the professional attendant—or from obstinacy, perverseness, or intemperance on the sufferer's own part.

"Some injuries, therefore, are fatal from peculiarity of constitution, or unfavourable circumstances of the patient; some prove fatal by accident; some are rendered fatal by the conjunction of disease; while others proceed to a fatal termination by neglect or improper treatment."—"It is, therefore, of great importance for us to inquire, not only what injuries prove inevitably fatal, but under what peculiar circumstances the slightest degrees of injury are followed with this result; in order that we may be able to say, in any particular case, whether the injury was sufficient to have caused death directly by the violence, or this event was the effect indirectly of other circumstances." 16.

### IV.—Medico-Legal Definitions of Wounds.

Every student knows the surgical division of wounds, into *incised, punctured, lacerated, contused,* and *gun-shot.*

In legal medicine the term is much more comprehensive, including *contusions, concussions, fractures, dislocations, sprains, burns,* or, in the words of our author:—"every local alteration of any part of the body, produced by violent means." 18.

Mr. Watson's chapter on this subject is acknowledgedly founded on the Treatise of Orfila—and adds nothing to the knowledge previously possessed by every tyro in the profession. Indeed, the four first chapters of his treatise may be said to be elementary merely, and to contain nothing that was not already before the public in previous publications. They possess, however, the merit of being concise and clear.

In the following chapters, which we now hasten to review, he treats his subject at length, and with a master-hand.

V.—Of Homicide by Injuries of the Nervous System, including Injuries of the Brain—Spinal-chord, and their Nervous Ramifications.

"The Study of Medicine" introduced a new era in the classification of diseases, arranging them according to the organs affected—at once a natural and easy order. Mr. Watson has carried Dr. Good's improvement into the study of Forensic medicine—and divides his subject "according to the organic systems affected by the injury or violence," which led to death—viz: the nervous, the circulating, the respiratory, the nutritive, and the generative systems.

Injuries of the nervous system frequently occur, and call for serious consideration, because a common cause of both sudden and lingering death in cases of homicide—and sometimes proving fatal, without leaving a trace behind in any alteration of structure.

These, he subdivides into four classes—(query, orders?) comprising:—α. Injuries of the tegumentary and bony parts of the cranium; β. of the brain; γ. of the spinal-chord; and, δ. of the nervous ramifications from the brain and spinal-chord.

α. *Injuries of the tegumentary and bony parts of the Cranium.*—Such cases are more or less frequently fatal, in proportion as they give rise to inflammation—whether phlegmonous or erysipelatous; the latter, often supervening upon a slight wound of the scalp—the former, sometimes extending to the membranes of the brain and even to the brain itself.

β. *Injuries of the Brain.*—Mr. Watson includes these under three *classes*—query, *genera?*—concussion or commotion, compression, and inflammation. In the first case, death is the effect of syncope, occasioned by a cessation in the heart's actions. In the second, of insensibility and coma, putting a stop to the respiration. In the third, of inflammatory and irritative fever—or of effusion, or of suppuration upon the surface of the brain causing compression.

In some cases there is no external lesion—yet, extreme internal disorder. In others the external injury is considerable. In some, death is the almost instant sequence; in others, the patient appears to have recovered—and is going about when suddenly seized with symptoms which hurry him to his grave.

Death—instant and sudden death from concussion may take place, without fracture of the skull—without extravasation of blood within the cranium—without perceptible organic lesion of any kind—and without furnishing any visible clue to a discovery of the immediate cause of death. A man fell from the mast-head on to the deck of a small frigate, on the East-India station—when brought below, he was labouring under symptoms of concussion. In a few hours he expired. The examination of his head, after death, afforded not the slightest trace * of any injury sustained by the brain. There was neither fracture nor extravasation.

In cases of fatal concussion of the brain from blows on the head, where the victim is generally knocked down and falls to the ground—it may be doubtful which caused the concussion—the fall or the blow: "When, in such cases, there is effusion of blood within the cranium, its situation, as corresponding with, or opposite to, an external mark of injury, from either the blow or the fall, may lead to an accurate conclusion." 36.

The doubts which have been raised in the criminal courts, respecting the possible fatality of blows on the head with the fist only, are completely set aside by the numerous cases which Mr. Watson adduces in proof of death having repeatedly taken place in consequence of such blows.

"In some of them it might be doubted whether the commotion and effusion of blood upon the brain were the effects of the blow, or (of) the fall received by the deceased when knocked down. But several circumstances tend to render it highly probable, if not certain, that the blow and not the fall was the cause. These are, first, such effects being very rarely if ever observed from similar falls without a violent blow at the same time; second, in most of the cases, the fall having been on soft ground; third, *the internal effusion of blood being generally found either at the place corresponding to the external mark of the blow, or on the side opposite to the external mark,* a circumstance which connects the effusion with the blow." 43.

Mr. Watson adduces the "insensibility, and failure of the *vis vitæ* which take place;—the hæmorrhage and softening of the brain observed in many fatal cases;—and the subsequent inflammation in cases of concussion, not immediately fatal," as all confirmatory of the view taken by him, that we are not to infer from the total absence in some cases of any observable lesion, the non-existence of such lesion, but the contrary. A proposition, the converse of which we incline to think is true—for insensibility and temporary failure of the vis vitæ occur in faintings, where there is confessedly no organic lesion in any part of the body; inflammations of other organs are not necessarily connected with previous lesions of those organs, and to infer that because hæmorrhage and softening of the brain are observed in some cases, something analogous, though invisible, *must* occur in all, is at once bad logic and indifferent pathology.

Extravasation of blood may destroy the patient who had sustained the violence of the shock of concussion, and was recovered therefrom. So may

---

* "Neither compression nor lesion, is necessary to the fatality of concussion, but it rarely happens that pure concussion induces a complete and continued suspension of the senses and voluntary motions. It is generally complicated in this case with extravasation or lesion of the brain, or its membranes, or both."—*Travers on Constitutional Irritation,* vol. i. p. 166.

inflammation, whether of the brain or its membranes—and so may the sequela of inflammation,—serous effusion upon the surface, and ramollissement of the substance of the brain.

The intervening time between the date of the injury, and its fatal termination is indefinitely various. In some cases, death being instant—in others, occurring after a few hours only have elapsed,—taking place after an interval of days in other cases—and in others again, being postponed for weeks, months, or even years. Pott and Abercrombie, have placed on record instances of life prolonged for weeks and months; and Serres mentions a case of concussion, in which the patient, after a blow on the back and side of the head, which stunned him at the time, became unsteady in his gait, and weak in his head; and subsequently, after about eighteen months, evinced increasing irritability of the nervous system,—and the well-marked progressive failure of his vital powers—for the left leg was, shortly after, paralyzed; the left arm became numb and weakened.

"On this account," to adopt the language of Dr. Gordon Smith, "the consideration of injuries from wounds, &c. in a medico-legal point of view, was formerly a matter of much more intricacy than it is now. A person inflicting violence upon another was held amenable for the consequences during a year and a day; a most inconvenient principle of responsibility, and one particularly calculated to create confusion, to involve the innocent, and in many cases to allow the guilty to escape. A person's death may be fairly traced to an injury, even though it may not take place for a much longer period than 366 days after that injury has been received; and on the other hand, one may die upon the receipt of a blow, and yet, that death has been thereby occasioned may not only be very doubtful, but even manifestly untrue."—*Principles of Forensic Medicine, 3rd edition, p.* 254-5.

The post-mortem appearances, in general obvious, though subject to variations, are, in severe cases, *extravasation of blood; separation of the dura mater from the cranium,* an appearance however which requires great caution in noticing it, as it may be imitated in the dead subject; *inflammatory effusion on the surface—effusion of fluid into the ventricles—and abscess, or softening of the brain.*

Extravasation of blood, however, so often happens within the cranium, irrespective of any external violence—as to lead to the important questions in cases of sudden death with suspicion of injury:—whether is such extravasation the result of violence? or of disease? or of strongly-excited passions and conflicting emotions? or of intoxication? or of the increased impetus given to the blood, in wrestling and struggling? or of the spontaneous rupture of a blood-vessel within the head?

*γ. Of Injuries of the Spinal Chord;—and δ, of Injuries of the Nervous Ramifications from the Brain and Spinal Chord.*—These, in general, prove fatal, and immediately or remotely so, according to their situation, extent and character. Death is immediate in cases of deep penetrating wounds, or where the upper part of the chord is injured;—in wounds, or compression of the medulla oblongata: in all which cases the mortality is the result of arrested respiration, and circulation. It is delayed, but only delayed, not averted, where the lesion is superficial, and situated at a lower point of the chord; such cases giving rise to paralysis and insensibility of the body below the injury, and there again occasioning, according to Mr. Travers, a feverish condition with depression, which, sooner or later, destroys the patient.

Sudden death is also a consequence of severe contusions inflicted on different parts of the body, such as blows on the pit of the stomach; and upon the abdomen, causing rupture of the viscera;—" contusions and lacerations of the extremities, particularly where the large joints have been crushed." The application of cold and heat alike, produce a similar result under certain circumstances. Professor Christian relates cases of sudden death from cold water received into the stomach of persons who were overheated at the time, and vice versâ, instances of extensive burns may be adduced to show a similar depressing effect produced by heat. In both, the cause of death is the same, a severe shock communicated to the sensorium through the nervous system, depriving it of its wonted power over the animal functions. Homicide, moreover, has been perpetrated by the application of escharotics to the surface, as well as by fire. Of the former, this country afforded a melancholy spectacle a few years ago in the case of St. John Long of unenviable notoriety. The Quack, Morrison, has demonstrated to the entire satisfaction of a believing public, if not to that of an acquitting jury, that such an effect may also be occasioned by the random administration internally of drastic drugs. The same effect has been noticed, in tropical climates, to follow a coup-de-soleil—and in all climates there have been cases of death by lightning.

"The depressing effect upon the action of the heart by a stroke of the sun, is similar to that produced by extensive burns. In such cases weakness of pulse, coldness, and other indications of collapse, have been observed. In other cases, and under more favorable circumstances, as relates to the vigour of the patient, and a shock of less intensity, an apoplectic state is produced, consisting of insensibility, with a full pulse and signs of turgidity of the bloodvessels about the head, accompanied with other symptoms of compression of the brain. Dissection in such cases has only shewn great increased fulness of the vessels within the head.

By the shock from lightning, individuals are sometimes instantly struck dead without any traces of its effects being apparent on the body. In some instances, however, there are marks like red stripes upon it; while in others, the viscera and other parts of the body have been found ruptured, the body peculiarly flaccid, and emitting a sulphurous or phosphoric odour.

When the individual struck by lightning is not killed on the spot, he is stunned, and the nervous system is affected from congestive apoplexy of the brain. From this he may die in seventeen or twenty-four hours, or if properly treated and the shock less severe, he may recover.

In cases of this kind, I am induced to consider the effects of the lightning owing to the short but intense impression, causing extreme excitement of the nervous system, suspending its functions, and thus bringing on a corresponding depression from which the individual does not recover. I have been led to draw this inference from the effects produced by a less violent stroke being those of excitement, and, in cases where the eyes are affected, of temporary suspension of the sensibility of the retina.

Different statements have been made as to the state of the blood in those who have been killed by lightning; some having represented that it does, others that it does not coagulate. In some cases it has been found issuing from the external openings of the body.

When, along with the appearances above-described, an individual is found dead after a thunder-storm, and particularly if metallic substances about him are found melted, or his clothes burnt, death may be attributed to the effects of lightning, if no other cause for death can be ascertained."—*Watson, on Homicide, p.* 83-4.

To conclude the present important and valuable section of the author's work in his own language:—

"In all cases of injuries of the head, the prognosis should be very guarded, and carefully deduced from a consideration of all the minute particulars of the case. Great caution and discretion are peculiarly necessary, and of vast importance in a medico-legal point of view: for, by the medical opinion given, the conduct of the public authorities is regulated, and upon it the fate of the prisoner depends. In some fatal cases, where no danger was at first apprehended, the prisoner has been tried for assault merely, and afterwards committed on a charge of murder. In other cases, the guilty person might escape altogether, from the authorities not being apprized of danger; while, on the contrary, the accused person might be doomed to the hardship of seizure and imprisonment without sufficient reason, be detained on groundless apprehensions, or prosecuted from an erroneous opinion given upon the case." 86.

### VI.—OF HOMICIDE BY INJURIES OF THE CIRCULATING SYSTEM.

These are, excessive hæmorrhage;—extravasated blood pressing upon vital organs and impeding their functions;—aneurismal tumors;—inflammation;—the entrance of air into the veins;—and, gangrene.

Death by hæmorrhage, from wounds of the neck, occurs less frequently in cases of homicide than of suicide. Yet murders have been committed in this way, "by wounds of the same kind, and in the same situation." It may occur without the larger vessels of the neck having been touched.

In wounds of the chest death sometimes takes place from the mechanical effect of the blood poured out from the wounded vessels into the cavities of the chest, and there interfering with the action of the heart and lungs.

"In this way the effusion of only a few ounces of blood into the pericardium proves instantly fatal, by its pressure upon the heart, which it surrounds. We have frequent opportunities of seeing illustrations of this, in the bursting of aneurisms of the arch of the aorta into the pericardium; as well as in the more rare cases of spontaneous bursting of the heart, and in some cases of wounds."—*Watson*, pp. 94, 95.

Wounds of the heart are not, of necessity, immediately fatal. Wounds of the aorta and pulmonary artery, are much more immediately and necessarily so. And still more so, wounds of the auricles, and coronary vessels. Sir George Ballingall, in his Outlines of Military Surgery (p. 327), goes the length of affirming that "*patients are known to have lived for some time after punctured wounds, even of the aorta!*" Hæmorrhage into the cavity of the chest, from wounds of the internal mammary, and intercostal arteries has, in some cases proved fatal. Hæmorrhage into the cavity of the abdomen and pelvis, from external wounds, has been attended with loss of life.

Death by hæmorrhage from wounds of the extremities has frequently occurred. The only cases we shall notice are those from wounds of the radial artery in performing the operation of blood-letting—and we do so for the benefit of our younger readers. Sir Astley Cooper places on record one of a man, who was bled in the hospital by a young gentleman, who, in doing so, penetrated the above artery; "thirty-seven ounces of blood were lost before he could succeed in stopping it," no very great deal except in peculiar states of the constitution, and not enough to destroy the patient: "in three days the pressure caused so much pain that the man requested it to be lightened; this was done and the bleeding returned; *at the end of the week* one of the surgeons deemed it prudent to secure the vessel, and he did so at the part where the wound had been made; the operation took an hour in performing, and it was excessively difficult to find the vessel. On the following day there were much irritation and inflammation, and on the tenth day from the accident he died!"—*Sir A. Cooper's Lectures*, p. 177.

Upon this case, it might have been made a question whether the carelessness of the young gentleman, or the tardiness of the old surgeon to repair the mischief done by his pupil, was most culpable. The death of their joint victim was an instance of homicide from improper treatment on the part of the professional attendant; a rare one, we both hope and believe, but not a solitary one we also know.

Air admitted into the veins, reaching the heart, and circulating to the brain, in that way produces death from wounds of the larger vessels, when the hæmorrhage from those vessels has not sufficed to destroy life. Mr. Watson cites only one case on the authority of Dr. Handyside, in support of this view—a case in itself extremely interesting but incomplete, and from its incompleteness, inadequate to the purpose for which it is quoted. It is one of suicide. A clergyman cuts his throat with both hands—one wound is two, the other four inches long—by the former, the trunk of the spinal accessory nerve, the conjoined origin of the occipital and posterior auris arteries, and the posterior facial vein are cut—yet "very little blood appeared to have been lost:"—by the latter, the transverse process of the atlas, the vertebral artery, the spinal accessory nerve, the external and internal jugular, the anterior and posterior facial veins, and the posterior auris artery are exposed. The facial artery slightly opened. Some globules of air were found to have entered into the internal jugular vein.

"By dividing the parts carefully under water, Dr. H. found air to escape from the cavities of the heart and large vessels connected with it, by which these parts had been moderately distended, but contained very little blood, and that in a frothy state. The veins of the liver and spleen also emitted air when they were divided under water."

The medico-legal questions connected with this section are,

1st. Did the individual die of hæmorrhage?

In order to prove the affirmative, it is requisite that the wound should have taken place during life—that the blood should have coagulated—and that such vessels were wounded as sufficiently to account for a fatal hæmorrhage—or that the wounded part was in a high state of vascularity.

2nd. Is the colour by which the clothes, floor, and other neighbouring substances are stained, the effect of blood? If of blood, it will be readily extracted by cold water, and the question decided by adding a solution of ammonia to the reddened water—the red colour of blood will alone remain unchanged by such solution. Or, "by boiling a watery solution of blood in a test-tube, the red is changed to a dirty greenish colour, and coagulated albuminous flakes appear mixed with the fluid."

3rd. In cases of mortal wounds of the heart, could the individuals have moved from the spot after receiving them, or must they have immediately fallen?

4th. If the patient did not fall immediately, would he, when he had fallen, be able to rise again?

### VII. Of Homicide by Injuries of the Respiratory System.

Death is produced in these cases by asphyxia—inflammation—and a combination of both.

Death by asphyxia may take place in a variety of ways, by suffocation, strangling, hanging, smothering, drowning, and intoxication.

Suffocation may be accidental, as when foreign bodies lodge in the larynx; or the head of a drunkard is placed in an unfavourable position, with his nose and mouth pressing upon the pillow; or it may be effected criminally, by securing the nostrils and mouth with the hands.

*Strangling* and *hanging* differ from each other only in the act of suspension in the last; both are occasioned by compression of the windpipe. Death by strangling, and also by hanging, may be accidental, self-inflicted, or the deed of a murderer. By the former it has been effected in some cases without leaving any marks on the neck—while, on the other hand, natural appearances have been mistaken for marks of strangulation. As death by hanging, we mean judicially, is likely to become a rare case in this country, we defer, for the present, any notice of the many curious facts brought together by Mr. Watson when considering the effects of that injury upon the human body. It is rarely the act of another, except as a punishment for crime:—the possibility of the case, however, has been shewn in the trial of a woman in Edinburgh, who fastened a ligature round her husband's neck, when asleep, and then drew him up. In cases of death by hanging, the medico-legal questions are:

First. Is it accidental—suicidal—or homicidal?

Secondly. Where a corpse is found hanging—was his death occasioned by hanging? or, was he first murdered and afterwards suspended?

For the answers to these highly important questions we must refer our readers to the work of Mr. Watson; regretting exceedingly that the length of this review, and the limited space remaining to us, preclude their transfer to our own pages.

Death by drowning is more frequently accidental, than death by either hanging or strangling—it is also a common form of suicide—and only rarely a homicidal act:

"Under peculiar circumstances, very little water suffices to cause death. Several instances have happened of individuals having been drowned by falling, when intoxicated, into a ditch, with their faces immersed in a puddle of a very shallow description. A short time ago, a gardener at Dalry, near Edinburgh, stooped his head into a large barrel, from which he wished to withdraw a pail of water. He overbalanced the lower part of his body and fell head-foremost into the barrel, where he remained, from being unable to extricate himself, and was found quite dead. In another case of this description, a man who lived in the Canongate, by a fall on the floor, was said to have got his head so fixed in a common iron pot, that he was suffocated." 147.

The questions to determine in cases of persons "found drowned" are:—

First—Whether the person died by drowning at all, or, was murdered first, and afterwards thrown into the water? and

Secondly—When death has been occasioned by drowning, was it suicidal or homicidal?

With respect to the first enquiry—what are the evidences of death by drowning? and are they always or altogether present? So far from being constant—persons have been drowned whose bodies presented no unusual appearances. And when present, they may in some cases, arise from other causes. Water in the air-passages—and froth conjoined with it—and water in a considerable quantity in the stomach, are strong presumptive proofs of death by drowning. The evidence upon which the proof of murder may be established is not medical—indeed, the science of forensic medicine supplies us with no means of answering the second inquiry.

Asphyxia may also be occasioned by *compression*, or *wounds of the* thorax or lungs—by injuries of and foreign bodies in, the larynx, trachea—and bronchial tubes. This last sort of accident is common to children, but has given rise to suspicion of homicide, and thereby endangered the innocent lives of others, on which account an examination of these parts becomes necessary in cases of death by asphyxia, from supposed external violence.

There is yet one more form of death by asphyxia, requiring to be noticed, especially in this gin and beer drinking age of the world, when more quarrels are fomented and concluded over a pot of "heavy wet" or a quartern of "blue ruin," aptly so called, than the readers even of the Sunday newspapers and slang chronicles have any conception of. Asphyxia from *excessive intoxication* is often accompanied with wounds and bruises giving rise to suspicion of murder, it is unnecessary to state that such concomitants may have been occasioned by accidental falls, without the infliction of any injury whatever by another person. Intoxication induces asphyxia by producing insensibility.

"This is well seen in cases of poisoning by opium, or, ardent spirits. When the patient becomes insensible, the breathing becomes remarkably slow, there being only two or three respirations in a minute; the pulse also becomes less frequent than natural; the surface of the body and the extremities become cold, and a state of complete collapse supervenes. From this state the patient may recover if the dose has not been very large, and when proper treatment is employed. But if the patient is without the necessary aid, and more especially when he is exposed to cold, death soon takes place, but in a gradual manner, by coma. In some individuals an apoplectic state is induced, in which the patient lives a few hours and then dies." 169.

The absence of spirits from the stomach is no proof against their being the cause of death; for, before the system can be effected, the spirits must be absorbed, and though the stomach be void of these, they will probably be found in the ventricles of the brain. The presence of spirits in the mouth, is, on the other hand, no evidence that death has been occasioned by the excessive use of them—they being frequently given by attendants to restore animation.

The concluding remarks of Mr. Watson, on death by intoxication contain matter for the consideration of society in general, and of governments in particular, which we can neither persuade ourselves to abridge, nor to omit:—

"I have repeatedly had occasion," he writes, "to observe the reckless want of consideration on the part of publicans, for any thing, but their own avaricious objects, in supplying young and intoxicated persons with excessive quantities of ardent spirits, quite regardless of the consequences to which it may lead. *In more than one case, I have known intoxicated persons admitted into these shops, from which, after having been supplied with spirits and paid their money, they have been inhumanly turned out at night to the street, and the door locked upon them.* IN THE MORNING THEY WERE FOUND DEAD, HAVING PERISHED, NO DOUBT, FROM EXCESSIVE INTOXICATION AND EXPOSURE TO COLD. When such cases as these, or of the regardless supply of spirits to young persons, occur and are made known to the public authorities, humanity certainly calls for some punishment upon the offender. The very common use of spirits, and other intoxicating liquors, without being followed by any fatal effects, has no doubt a tendency either to prevent or banish from the mind any idea of danger from the practice, yet their poisonous and fatal effects, when taken in excessive quantities, are not less certain, and ought to be generally made known to the public. It is not uncommon for the lower orders to ascribe the fatal effects of spirits to what they please to term *'bad drink,'* when, if I mistake not, its only and greatest fault is, its having been too strong, and considered too good by its infatuated victims. Hence arises the very singular circumstance of the poisonous effects of an article of luxury in common use, and of a crime arising from its being given in improper quantities." 177.

Contusions and fractures of the ribs, and wounds of the pleura and lungs, also prove fatal from their depressing the vital powers by the violence of the shock which the constitution receives from them, or from their inducing inflammatory fever. Such injuries are more or less mortal, as the subjects of them are more or less temperate, and in sound health; these modifying circumstances, requiring, of course, to be taken into consideration by the medical jurist, in every case of death after injuries like the above. Numerous cases are on record of the immense violence sustainable by the chest as well as other parts of the human body without its issuing in death. The case of the young nobleman, whose heart the celebrated Harvey handled in the King's presence through an open wound of the chest, is one of the most remarkable, and we have ourselves witnessed cases of thoracic injury from gun-shot wounds, where ribs have been broken and lungs lacerated, with very considerable subsequent inflammation and fever, but with ultimate recovery. If we err not, Mr. Rutherford Alcock had several such cases under his care in Spain, the recovery of which was principally owing to the unremitted care and attention of their professional attendants.

### VIII. OF HOMICIDE BY INJURIES OF THE NUTRITIVE SYSTEM, AND ORGANS OF REPRODUCTION.

This may take place *without lesion of the internal organs, and without inflammation,* as in the case of blows inflicted on the region of the stomach—and *with lesion of the internal organs, but without inflammation.*

To adopt the language of Mr. Travers, "certain forms of mortal injury are productive of sudden, excruciating, and unremitting pain, such as ruptures of the stomach, gall, and urinary bladders. Death ensues in these cases many hours sooner than where the pain is less intense, and before the morbid changes which take place in consequence of the injury are so far established as to make it credible that the result is to be ascribed to their influence." Other injuries prove fatal *from supervening inflammation,* without lesion of internal parts; and others *by inflammation from lesion of internal parts,* generally rapid in its progress, and productive not only of increased vascularity, but also of inflammatory effusion "consisting of bloody serous fluid and flakes of yellow matter, both floating in the fluid, and coating the peritoneal surfaces. A feeble pulse, sense of depression, look of exhaustion, a cold surface and colder extremities with collapse, evince a reduced action of the heart, the effect of the shock communicated to the nervous system—and this is perhaps the most striking feature in these cases.

But here we must bring our notice of Mr. Watson's very valuable work to a close. There remain four chapters untouched: the IXth, "Of the circumstances which modify the Medico-legal Character of Wounds and other Injuries." Xth. "Of the Medico-legal Examination of Wounds." XIth. "Of Concealment of Pregnancy and Infanticide," and, XIIth. "Of the Exculpatory Plea of Insanity in Cases of Homicide." Nor have we left ourselves room to notice them further than by a word of general commendation, applicable to each and merited by all.

We have seldom met with a work of greater merit than that now reviewed. It is clear, concise, compendious, and rich in facts. We hail its appearance as the dawn of a better day in medical literature, for there is not a particle of book-making in the whole volume. We recommend it to the perusal of our readers as full of important matter—the more attentive their perusal, the higher will be their recompence.

But, as a review without fault-finding, would be as great an anomaly in the art of criticism as a book without fault in the craft of authorship, we will just remind our author that in some places his writing is loose, not self-consistent, and even contradictory—while in others, he occasionally, although very rarely, cites cases to prove a proposition, which fail in doing so, being impertinent and irrelevant.

We now take our leave of this experienced surgeon and talented writer, with an expression of unfeigned respect and gratitude for one of the most important contributions to the science of medical jurisprudence with which we are acquainted. And as we are persuaded his object has been, and is, to promote the cultivation of that science, among the present and future generations of medical jurists, we would take the liberty of recommending that his next edition be printed in another and cheaper form for the benefit of students. And one way of effecting this might be, by printing all the cases, 238 in number, in small type, and such of them as were previously before the profession in other publications, in a smaller type still. The expense of the volume would, in that way, be diminished one half—and its circulation increased four-fold. We earnestly recommend this plan because we are sincerely of opinion that it deserves to be on the table of every medical jurist, and in the hand of every medical student in the three kingdoms.

